# „Enhanced Recovery after Intensive Care – ERIC“

**DOI:** 10.1007/s00101-020-00863-x

**Published:** 2020-10-07

**Authors:** N. Paul, J. J. Grunow, B. Weiß, C. Spies

**Affiliations:** Klinik für Anästhesiologie mit Schwerpunkt operative Intensivmedizin, Charité – Universitätsmedizin Berlin, corporate member of Freie Universität Berlin, Humboldt-Universität zu Berlin, and Berlin Institute of Health, Augustenburger Platz 1, 13353 Berlin, Deutschland

## Hintergrund und Hypothesen

Jährlich werden in Deutschland mehr als 2 Mio. Patienten auf eine Intensivstation (ITS) aufgenommen [1], gleichzeitig überleben immer mehr Patienten ihre Intensivbehandlung [2]. Viele Überlebende zeigen Beeinträchtigungen ihrer kognitiven Funktionen, psychischen Gesundheit oder Mobilität, zusammengefasst als „Post-Intensive Care Syndrome“ (PICS) [3, 4]. Zur Verhinderung dieser Langzeitfolgen ist eine hohe Behandlungsqualität während der intensivmedizinischen Behandlung von großer Wichtigkeit. Hierfür hat die Deutsche Interdisziplinäre Vereinigung für Intensiv- und Notfallmedizin (DIVI) Qualitätsindikatoren (QIs) in dritter Auflage festgelegt, deren Umsetzung zur Verhütung von Langzeitschäden beitragen kann [5].

## Details der Studie

**Figure 1:**
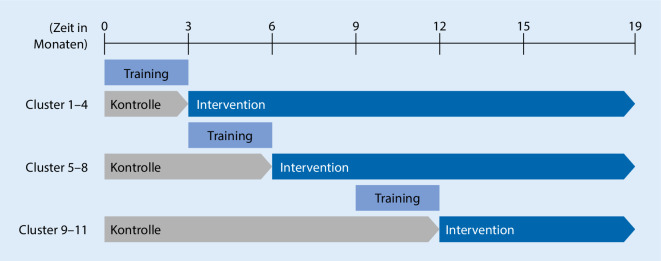

**Figure 2:**
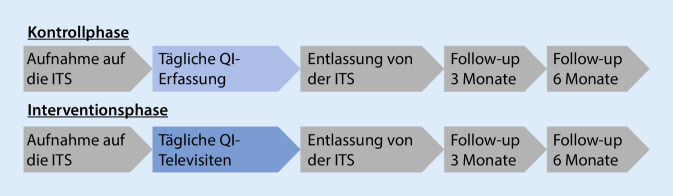

Enhanced Recovery after Intensive Care (ERIC) beschreibt eine neue Versorgungsform in der Intensivmedizin, bei der Intensivpatienten in der Region Berlin/Brandenburg telemedizinisch behandelt und in einem angeschlossenen „Case-Care Management“ versorgt werden [6]. Es ist eine multizentrische, clusterrandomisierte, kontrollierte Studie im Stepped Wedge Design. 11 Cluster (15 ITS) beginnen in der Kontrollphase, in der Patienten konventionelle Intensivtherapie erhalten, jedoch täglich die Erfüllung (ja/nein) von 8 QIs der DIVI zur akut-intensivmedizinischen Versorgung dokumentiert wird [5]. An den im Studienprotokoll festgelegten Zeitpunkten wechseln die Cluster in die Interventionsphase (Abb. 1). Dort erhalten Patienten tägliche, telemedizinische Visiten durch erfahrene Intensivmediziner der Tele-ICU der Charité – Universitätsmedizin Berlin. Fokus der Visiten ist die Erfüllung der QIs der DIVI [5], deren Einhaltung täglich erfasst wird. Drei Monate vor Beginn der Televisiten durchlaufen teilnehmende Zentren ein als Blended Learning konzipiertes Training zu den QIs und zum Umgang mit dem telemedizinischen Device. Drei und sechs Monate nach der Entlassung von der ITS erhalten Patienten in Zusammenarbeit mit den HausärztInnen eine strukturierte Nachsorge zu möglichen Langzeitfolgen des ITS-Aufenthalts (Abb. 2; [7]).

Einschlusskriterien sind ein Alter von mindestens 18 Jahren, ein ITS-Aufenthalt von mindestens 24 h und ein gesetzlicher Krankenversicherungsstatus. Primäre Endpunkte sind die tägliche Erfüllung der 8 QIs der DIVI; sekundäre Endpunkte sind u. a. (bis zu 6 Monate nach ITS-Entlassung): Sterblichkeit, mentale Gesundheit, kognitive Funktionen, Mobilität und gesundheitsbezogene Lebensqualität.

## Statistik

Unter Berücksichtigung von 8 binären, gleichwertigen co-primären Endpunkten und entsprechender Bonferroni-Korrektur, einer angenommenen Steigerung der QI-Adhärenz von mindestens 10 % sowie der Korrelation von Patienten innerhalb eines Clusters beträgt die errechnete Fallzahl 1431 Patienten. Es ist geplant, die Analyse nach dem Intention-to-Treat-Prinzip durchzuführen, und die einzelnen QIs werden bei einem Signifikanzlevel von 0,625 % verglichen.

## Ethik

Die Ethikkommissionen der Charité – Universitätsmedizin Berlin (EA1/006/18) und der Medizinischen Hochschule Brandenburg Theodor Fontane (Z-01-20180828) stimmten dem Vorhaben zu.

## Meilensteine

Der Patienteneinschluss begann im September 2018 und konnte im März 2020 unter Erreichen der Fallzahl beendet werden. Die Nachverfolgungen im Rahmen des Case-Care Managements werden im Oktober 2020 abgeschlossen und das Evaluationsergebnis wird im Frühjahr 2021 vorliegen.

## Studiengruppe/Expertise

### Konsortialführerin

- Klinik für Anästhesiologie mit Schwerpunkt operative Intensivmedizin, Charité – Universitätsmedizin Berlin (Studienleiterin: Univ.-Prof. Dr. med. Claudia Spies)

### Konsortium

- Fachgebiet Management im Gesundheitswesen, Technische Universität Berlin (Prof. Dr. Reinhard Busse)
- Institut für medizinische Informationsverarbeitung, Biometrie und Epidemiologie (IBE), Ludwig-Maximilians-Universität München (Univ.-Prof. Dr. Ulrich Mansmann)
- Klinikgruppe Ernst von Bergmann (Dr. Simone Rosseau)
- BARMER (Dr. Ursula Marschall)
- Fraunhofer-Institut für Offene Kommunikationssysteme FOKUS (Ben Kraufmann)

## Sponsor/Finanzierung

Innovationsfonds des Innovationsausschusses des Gemeinsamen Bundesausschusses (Fördernummer: 01NVF16011).

## Link zur Studienbeschreibung der DGAI

ClinicalTrials.gov: NCT03671447 (verfügbar unter: https://www.clinicaltrials.gov/ct2/show/NCT03671447. Zugegriffen: 29.09.2020).
